# Relevance of the pyroptosis-related inflammasome drug targets in the *Chuanxiong* to improve diabetic nephropathy

**DOI:** 10.1186/s10020-022-00567-5

**Published:** 2022-11-18

**Authors:** ChangYan Li, JingYuan Ma, Niroj Mali, Le Zhang, Tao Wei, LuYao Shi, Fang Liu, Fan WenXing, Jing Yang

**Affiliations:** 1 Department of Nephrology, The First Affiliated Hospital of Kunming Medical University, No. 295, Xichang Road, Kunming, 650032 Yunnan China; 2 Institute for Integrative Genome Biology, University of California Riverside, Riverside, CA 92521 USA; 3 Kunming Medical University, Kunming, 650500 Yunnan China; 4 Department of Nephrology, West China Hospital, Sichuan University, Chengdu, 610041 Sichuan China

**Keywords:** *Chuanxiong*, WGCNA, Pyroptosis, Diabetic nephropathy

## Abstract

**Background:**

A chronic inflammatory disease caused by disturbances in metabolism, diabetic nephropathy (DN) is a chronic inflammatory disease. Pyroptosis is a novel form of programmed cell death in many inflammation-related diseases, including DN. Therefore, pyroptosis could be a promising target for DN therapy.

**Methods:**

To get the components and pharmacodynamic targets of *Chuanxiong*, we identified by searching TCMID, TCMSP, ETCM and HERB databases. Then, from the Molecular Signatures Database (MSigDB) and Gene Ontology (GO) database, pyroptosis genes were collected. Identification of critical genes in DN by bioinformatics analysis and then using the ConsensusClusterPlus package to divide the express data of diff genes into some subgroups with different levels of pyroptosis; the WGCNA machine algorithm was used to simulate the mechanism *Chuanxiong* improving DN.

**Results:**

In this study, we found DHCR24, ANXA1, HMOX1, CDH13, ALDH1A1, LTF, CHI3L1, CACNB2, and MTHFD2 interacted with the diff genes of DN. We used GSE96804 as a validation set to evaluate the changes of APIP, CASP6, CHMP2B, CYCS, DPP8, and TP53 in four different cell proapoptotic states. WGCNA analysis showed that DHCR24, CHI3L1, and CACNB2 had significant changes in different cell proapoptotic levels. In the experimental stage, we also confirmed that the active ingredients of *Chuanxiong* could improve the inflammatory state and the levels of pyroptosis under high glucose.

**Conclusion:**

The improvement of DN by *Chuanxiong* is related to the change of pyroptosis.

## Background

Diabetic nephropathy (DN) is a significant complication of diabetes mellitus and is the most common cause of end-stage kidney failure (Wonnacott et al. 2022); it is characterized by glomerular hypertrophy, mesangial matrix accumulation, mesangial cell proliferation, glomerular sclerosis, and proteinuria (Dai et al. 2021a; Wang et al. 2020; Yang et al. 2022). As a microvascular response, DN is involved in the majority regulation of inflammation and immunity (Li et al. 2020b; Zhang et al. 2022a). However, the mechanism of inflammation regulation has not been deeply studied. In recent years, evidence supports cell pyroptosis in the pathogenesis of DN inflammation. Furthermore, gasdermin D (GSDMD) stimulation of plasma membrane pore formation, cell expansion, and rapid lysis, as well as the production of proinflammatory cytokine interleukin (IL)-1β and IL-18 (Burdette et al., 2021; Rühl et al. 2018), which trigger a cellular inflammatory response, there are potential advantages to this approach in drug development.

The active ingredients of *Chuanxiong* include alkaloid, tetramethylpyrazine (TMP), ferulic acid, canola alcohol, octadecanoic acid, and so on (Wang et al. 2022b). TMP is one of the most important active components in *Chuanxiong* and has been used in oriental medicine for a long time to treat stroke and cardiovascular diseases (Zhang et al. 2022b). Besides, TMP is a compound originally isolated from the rhizome of *Chuanxiong*. Its remarkable vasodilation and antihypertensive effects also impact antioxidation, correcting hypercoagulability and promoting vascular recanalization (Lin et al. 2022). It has been reported that TMP can increase phosphorylated Akt (p-Akt) and B-cell lymphoma-2 (Bcl-2) and decrease the expression of phosphorylated glycogen synthase kinase-3β (p-GSK-3β), Bcl-2-associated X protein (Bax) and Cleaved caspase-3 by activating the Akt signaling pathway of Akt signaling (Rai et al. 2019; Zhu et al. 2022), improvement of metabolic markers of diabetes, and inhibiting of oxidative stress improved DN in rats. The damage of podocytes in a high glucose environment can affect the function of the glomerular capillary and lead to the destruction of renal function. *Chuanxiong* has been used as a vasoactive drug for a long time, with TMP as a representative. However, the mechanism of *Chuanxiong* affecting vascular activity is not precise. Our study will clarify this mechanism of *Chuanxiong* based on network analysis of *Chuanxiong* therapeutic targets to construct the relationship between DN and the regulation of inflammation-related mechanisms of *Chuanxiong* and to further clarify the relationship between the improvement of inflammatory response and podocyte pyroptosis.

## Methods

### Collection of drug-target data of Chuanxiong

The keywords "*Chuanxiong*" were searched in the TCMSP, TCMP, ETCM and HERB databases, with the target information of *Chuanxiong* was collected. We downloaded the GSE30122 and GSE96804 from the NCBI-GEO database. The GSE30122 of DN data was used as the experimental data set for this study; consisting of 9 glomerular samples from DN patients and 26 normal controls. Besides, GSE96804 consisting of 41 glomerular samples from DN patients and 20 normal controls. Using the “limma” R package to obtaining DN-related differential genes under the conditions of Logfoldchange = 1, adjust P = 0.05. In addition, by searching MSIGDB and Gene Ontology Database to collect pyroptosis genes.

### Core target genes mediate protein-protein interaction (PPI) networks

In this study, using protein-protein interaction in the STRING database to explain the interaction between the active chemical components of *Chuanxiong* and the core genes, cytoscape 3.7.2 software was used to optimize the results.

### Construct the DN model of different pyroptosis state groups

Consensus clustering is an unsupervised clustering method based on re-sampling to verify the rationality of clustering. The primary purpose of this study is to evaluate the stability of clustering. Six focus-related genes, APIP, CASP6, CHMP2B, CYCS, DPP8 and TP53, were grouped, and K = 4 was the best number of clusters. In addition, the rows and columns of the matrix represent samples, and the cluster analysis results are similarly distinguished by blue and white, with the correlation of the cluster score from 0(non-cluster) to 1(cluster). The bar graph between the tree and the thermograph is a separate category.

### Co-expression analysis

We used R (version 4.0.3, X64) software installed WGCNA package to analyze the co-expression of differentially expressed genes. In the calculation, we use multi-thread work, set the power function index range to 1:20, calculate the dependency matrix: “ai, j = | Cor (i, J) | β,“ determine the best β value and then calculate the module gene number according to the similarity between two genes by topological overlap (TOM), then the hierarchical clustering analysis is carried out, through the transformation of the dissimilarity between genes for obtained the gene clustering tree. Meanwhile, in the dynamic hybrid cutting algorithm to cut the gene clustering tree, the number of genes in each cluster group is at least 50, so we can divide the identical genes into the same modules. Finally, we merge very similar modules through module exigencies (ME).

### Western blot

Add the primary antibody and the corresponding secondary antibody under appropriate conditions. The primary antibody was placed in a refrigerator at 4 °C overnight. After 24 h, the corresponding secondary antibody reaction was added for 2 h, followed by three times of intermittent washing with PBS solution. See previous research for details.

### Quantitative real-time polymerase chain reaction (qPCR)

Total RNA was extracted from renal cortical tissue using TRIzol reagent (Tiangen Biotech Co., Ltd., Beijing, China) and reversely transcribed into cDNA using the First Strand cDNA Synthesis Kit (Tiangen Biotech Co., Ltd., Beijing, China). qPCR analysis was performed using the Heff TM qPCR SYBR® Green Master Mix Kit (Yepsen Biotech Co., Ltd., Shanghai, China) and a real-time fluorescence quantitative PCR system (MA-6000, Molarray, Suzhou, China). The relative mRNA quantification was calculated using the 2-ΔΔCt method. Target mRNA expression was normalized to the housekeeping gene Glyceraldehyde 3-phosphate dehydrogenase (GAPDH) and presented as fold change compared with the control group. Primer sequences are listed in Table 1.

**Table 1:** Sequence of quantitative real-time polymerase chain reaction (qPCR)

Ollgo name		Sequence	Length (bp)
IL-1β	Forward Reverse	ttc gag gca caa ggc aca a cca tca ttt cac tgg cga gc	19 20
NF-κB	Forward Reverse	gtc gca tcc aca gtt tcc ag gca ttc agg tcg tag tcc cc	20 20
GAPDH	Forward	ggc acc act act tca gag acc aag g	25
	Reverse	aca cga ggg cac aga aag caa tag	24
NLRP3	Forward	aag aca ggc agg cag cac aat g	22
	Reverse	ggc aga aga gga aag gag cac ata g	25
IL-18	Forward	gac agg gaa gag gag gag atg aga g	25
	Reverse	gaa gca ggt gag agt aag cga agg	24

### Pyroptotic cell death assay-lactate dehydrogenase (LDH) release assay

The LDH release was detected by CytoTox 96® Non-Radioactive Cytotoxicity Assay (Promega Corporation, G1780, Madison, USA). According to the instruction, the substrate mixture was prepared by adding 50 mL to each sample pore Incubate the board at room temperature for 30 min, away from light. The absorbance was measured at 490 nm with a microplate reader.

### Cell transfection

The logarithmic human renal podocytes were seeded into 6-well plates and divided into miR-NC group and miR-NLRP3 group. NLRP3 knockdown vector sequences 2638,3066, and 1208 were purchased from Gene Pharma, and sequences (5′-3′) are as follows:

**Table:** 

NLRP3-2638	S:GCCAGCUGGAALUGUUCUATT
	AS:UAGAACAAUUCCAGCUGGCTT
NLRP3-3066	S: GGCUGUAACAUUCGGAGAUTT
	AS: AUCUCCGAAUGUUACAGCCTT
NLRP3-1208	S:GUGCAUUGAAGACAGGAAUTT
	AS: AUUCCUGUCUUCAAUGCACTT

After transfection, the cells were cultured in high glucose for 48 h and then collected for WB and q-PCR.

### Statistical methods

All the data were expressed as means ± standard error of the mean (SEM). GraphPad Prism 6.0 software was used for statistical analysis. Comparisons between two groups were performed using Student’s t-test. One-way analysis of variance (ANOVA) followed by Tukey’s post hoc test was performed when comparisons among multiple groups. Each experiment was repeated three times. A P value < 0.05 was considered statistically significant.

## Results

### Analyze the correlation between disease and drug target

**Fig. 1:**
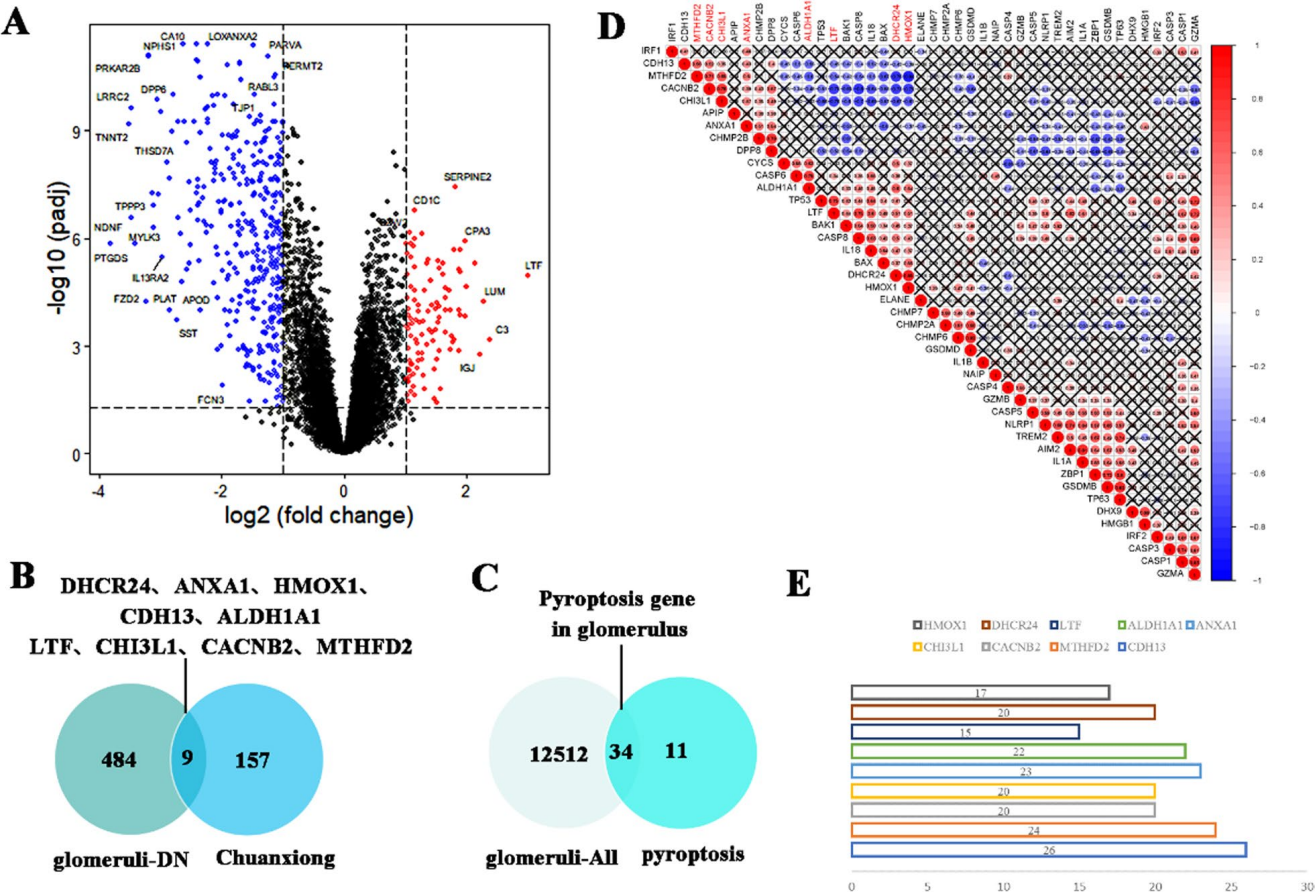
**A** Diff genes of DN by using the limma package (LogFC = 1, *P* < 0.05); **B**, **C** screening of drug target gene; **D** drug and disease target interaction for DN; **E** the 9 drug target genes were sequenced according to the number of correlation degree with pyroptosis genes *P < 0.05; **P < 0.01; ***P < 0.001

DN is chronic microangiopathy caused by diabetes; inflammation and immune responses are vital regulators that influence its development. Pyroptosis plays a regulatory role in diabetes-induced diseases as a form of necrotic apoptosis. This study, Uses GSE30122 samples to analyze the differential genes in DN (Fig. 1A). Meanwhile, targets of *Chuanxiong* active ingredients were collected through ETCM, TCMID, TCMP, ZYCB, and other databases to determine the relationship between target drugs and diseases. The results indicate that DHCR24, ANXA1, HMOX1, CDH13, ALDH1A1, LTF, CHI3L1, CACNB2 and MTHFD2 may be involved in the regulation of DN (Fig. 1B, C). Besides, for constructing the correlation between nine genes and glomerular pyroptosis genes. To evaluate the efficacy target of *Chuanxiong* and pyroptosis genes in glomerular cells by the Corrplot function. The above results suggest that nine genes are associated with the pyroptosis, which jointly affects the occurrence and development of DN. As the top targets, CDH13, MTHFD2, ANXA1 and ALDH1A1 have 22 to 26 genes involved in the regulation of pyroptosis by drug targets, respectively (Fig. 1D, E). Combined with our findings, there is a correlation between the drug target of *Chuanxiong* and the differential genes of DN. The protection of *Chuanxiong* to improve DN may be related to regulating cell pyroptosis.

### *Chuanxiong* influences pyroptosis to improve DN

**Fig. 2:**
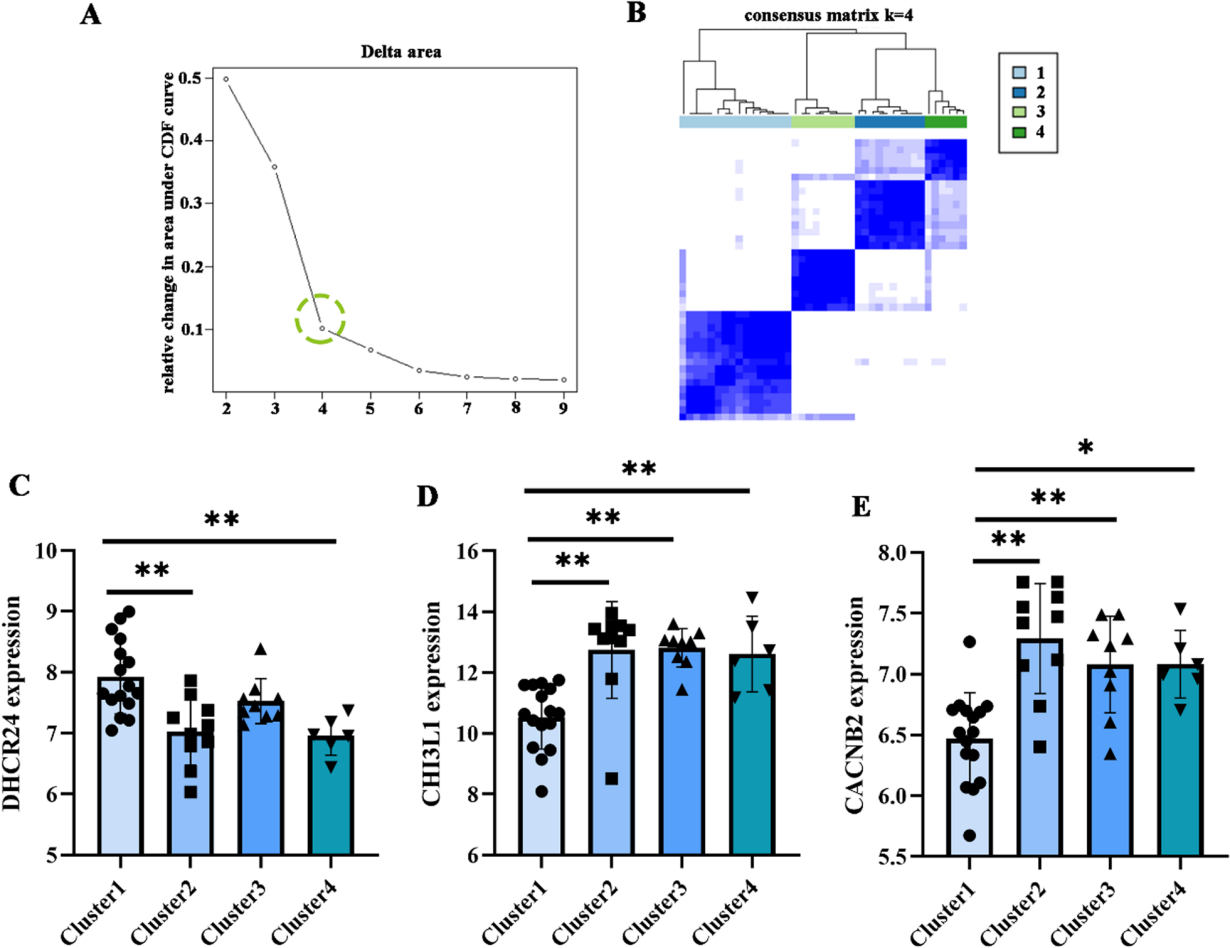
**A**, **B** Grouping the validation dataset based on the expression of DN-related pyroptosis genes; **C–E** analysis of differential gene expression in different pyroptotic status groups. *P < 0.05; **P < 0.01;***P < 0.001.

As a type of inflammatory death, studies have shown that microvascular lesions, including DN, are regulated by an inflammatory response involving complement activation and immune response. The relevant evidence indicated that the active components of *Chuanxiong* could pass TLR4/TRAF6/ NF-κB/NLRP3/Caspase-1 and TLR4/Caspase-8/Caspase-3 signal pathway to improve the state of cell pyroptosis, which proves that *Chuanxiong* can regulate the process of cell pyroptosis. To simulate whether the regulation of DN by *Chuanxiong*. GSE96804 investigated DN’s pathological mechanism, including standard controls (20) and DN (41). According to the expression of 6 focused genes (APIP, CASP6, CHMP2B, CYCS, DPP8 and TP53) by cluster plus algorithm, we divided the DN samples of GSE96804 into four subgroups (Fig. 2A, B). Furthermore, DHCR24, ANXA1, HMOX1, CDH13, ALDH1A1, LTF, CHI3L1, CACNB2, and MTHFD2 in different pyroptosis states of the disease. The results showed that the target sites (DHCR24, CHI3L1, CACNB2) of *Chuanxiong* differed in different groups (Fig. 2C–E).

### Chuanxiong has a pyroptosis regulatory target for DN

**Fig. 3:**
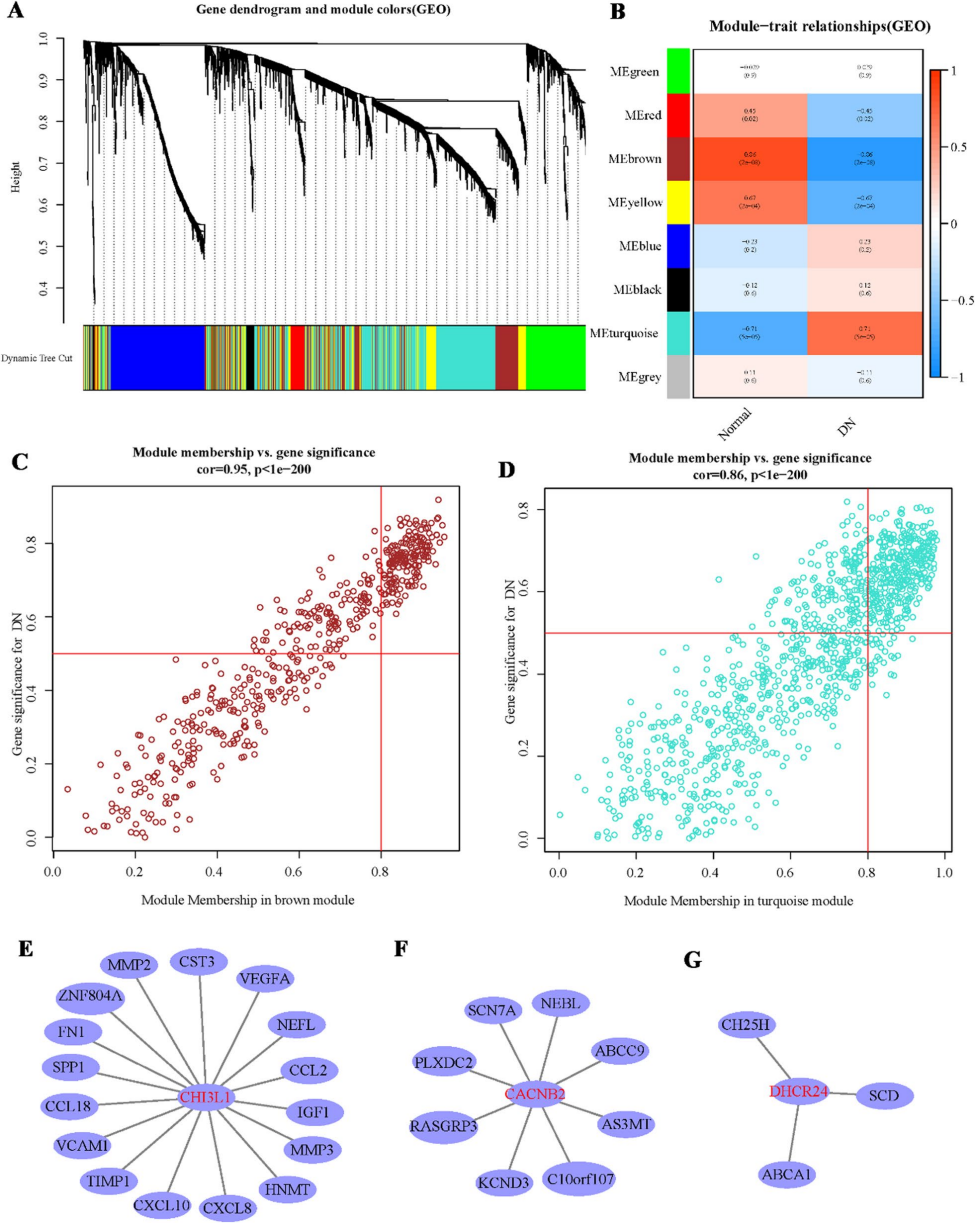
**A–D** WGCNA algorithm sifted out DHCR24, CHI3L1, and CACNB2, which have mutual relations; **E–G** the interaction network of DHCR24, CHI3L1, and CACNB2 with modular genes. *P < 0.05; **P < 0.01; ***P < 0.001.

During the analysis, the target sites of *Chuanxiong*. DHCR24, CHI3L1, and CACNB2 take into the regulation of DN, but the mode of regulation was not transparent. Besides, WGCNA was used to analyze the regulatory relationship of DHCR24, CHI3L1, and CACNB2 in four subgroups with different cell scorch levels (Fig. 3A–D); there was a network of interaction between the subsection 1 and 2, and the standard control in the brown module and the Sapphire module. The target sites of DN were DHCR24, CHI3L1, and CACNB2 (Fig. 3E–G); this further confirmed that the drug and disease target network of *Chuanxiong* and DN could play a protective role in the improvement of cell pyroptosis.

### Pyroptosis is a potential target of *Chuanxiong* for improving DN

**Fig. 4:**
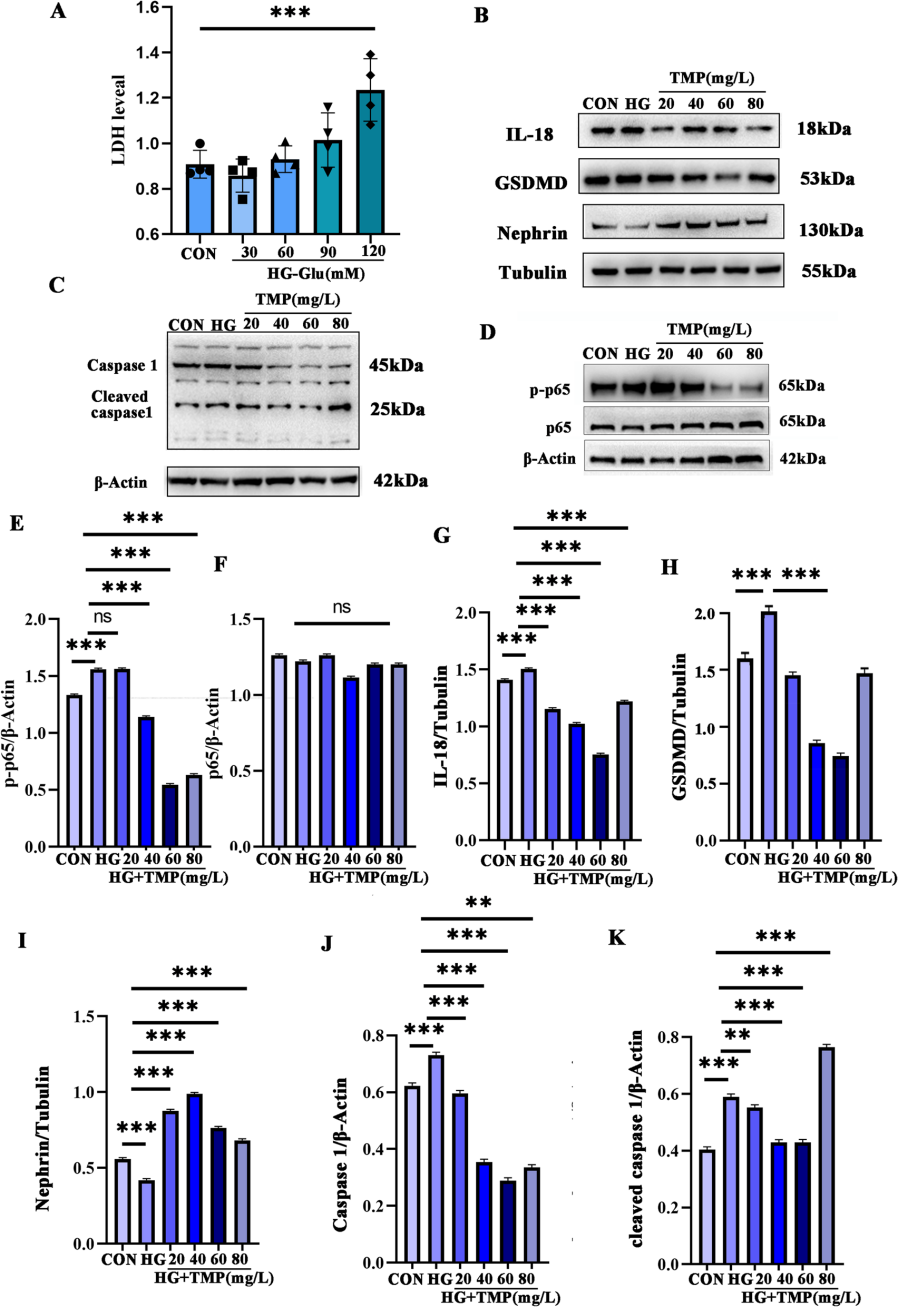
**A** LDH releases after podocyte injury under a high glucose environment; **B–D** evaluation of the improvement of pyroptosis and inflammatory cellular state by the active ingredient of *Chuanxiong*; **E**–**K** statistical analysis of pyroptosis and inflammation-related protein expression. *P < 0.05; **P < 0.01; ***P < 0.001.

Analysis of transcriptome data of target genes of active components of *Chuanxiong* and drug efficacy provided an essential link for improving the regulation of DN. Rundong Jiang confirmed that TMP, the active ingredient of *Chuanxiong*, can inhibit NF-κB/NLRP3/Caspase-1 signaling and improve and pyroptosis of macrophages, which indicates that *Chuanxiong* can improve the process of inflammatory response. Studies propose that the effector protein of pyroptosis is GSDMD, and Caspase1 is involved in regulating inflammation in regulating pyroptosis. In this part of the demonstration experiment, the high-glucose environment can accelerate the release of LDH when podocyte injury (Fig. 4A). *Chuanxiong* intervention can prohibit GSDMD up-regulated lead by high glucose, with Caspase1 activated (Fig. 4C, J, K); when Caspase1 activation can affect the membrane pores GSDMD. Formation promotes the release of IL-18, and TMP helps to slow down this process (Fig. 4B, G). There is related to changes in the pyroptotic state. Moreover, to interpret the pharmacological effect of *Chuanxiong* on mitigation injury to combine with DN. Then, in the high glucose state, the structure of podocytes is damaged, and the expression of the membrane structural protein Nephrin is significantly down-regulated.

After high glucose intervention in podocyte, we found that Nephrin expression was improved concentration-dependent by TMP intervention (Fig. 4B, I). Our study detected the expression of resident inflammatory initiator IL-18 in macrophages and found that the expression of IL-18 was associated with the activation of NF-κB signaling. Under the same high glucose environment, damage to the podocyte brings about the up-regulated expression of IL-18. p-P65 increased in podocytes induced by high glucose (Fig. 4D, E), suggesting that high glucose can cause podocyte inflammation.

### TMP, an active component of *Chuanxiong*, inhibits the phosphorylation of NF-κB protein and affects podocyte pyroptosis

**Fig. 5:**
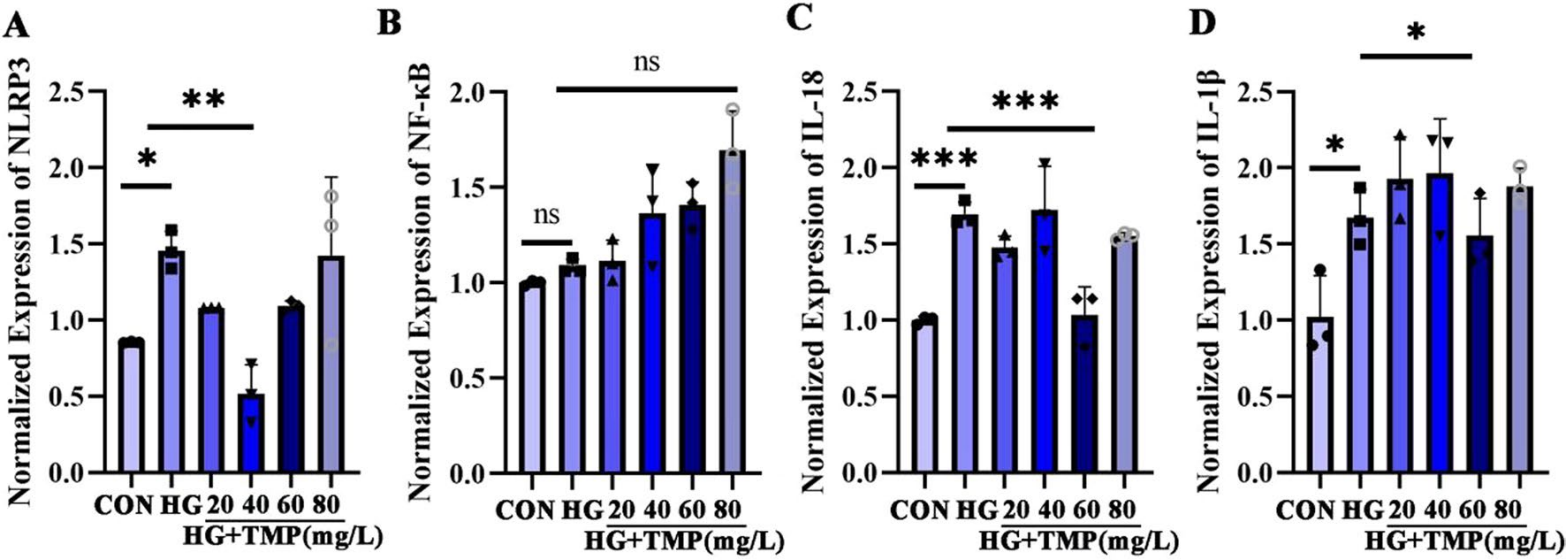
**A–D** To evaluate the improvement effect of *Chuanxiong* active components on inflammatory factors at transcription level. *P < 0.05; **P < 0.01; ***P < 0.001

**Fig. 6:**
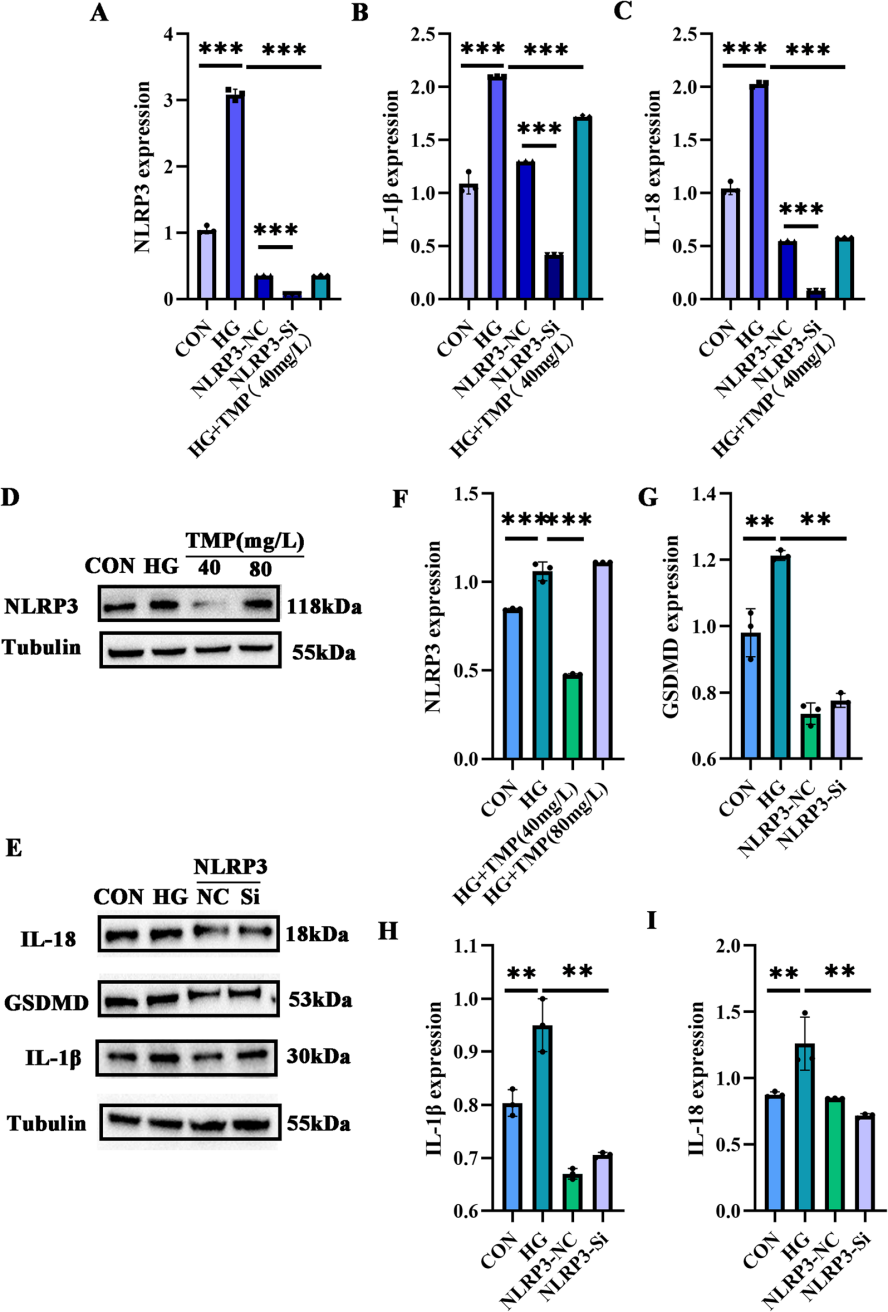
**A–C** NLRP3-silence podocyte model construction, NLRP3, IL-1β, and IL-18 gene expression profiles after TMP intervention and NLRP3 knockdown models; **D**, **E** TMP at a relatively optimal concentration of intervention or NLRP3 silence; The expression of GSDMD, NLRP3, IL-1β and IL-18. F-I: the protein expression of GSDMD, NLRP3, IL-1β, and IL-18 were analyzed statistically. *P < 0.05; **P < 0.01; ***P < 0.001

The results showed that NLRP3 increased in the podocytes under a high glucose environment and decreased concentration-dependent after TMP intervention. The effect of TMP was most significant at the dose of 40 mg/L, while that of TMP at the dose of 80 mg/L was most significant. NLRP3 elevation allows for the emergence of a drug toxic effect that increases the inflammatory response (Fig. 5A). NF-κB did not change significantly at the transcriptional level (Fig. 5B), which is consistent with previous protein findings that NF-κB is not activated at the transcriptional level. In contrast to repressed expression at the transcriptome level, NF-κB was phosphorylated to p-P65 at the protein level; after we intervened with TMP, p-P65 expression was significantly reduced in the range of 40–80 mg/L concentrations. It can therefore be hypothesized that NF-κB interacts with NLRP3 at the protein level, resulting in phosphorylation of NF-κB at the protein level being activated to p-P65. Thus, regulation of IL-1β and IL-18 further triggers GSDMD-mediated inflammatory cell necrosis leading to pyroptosis production; at the same time, TMP intervention can ameliorate NLRP3-mediated apoptosis. Besides, the expression of NLRP3, IL-1β and IL-18 were significantly down-regulated by TMP or at NLRP3 silent expression podocyte model (*P* < 0.05) (Fig. 6A–C); NLRP3 expression was significantly repressed compared with the high glucose model group (Fig. 6D). When the NLRP3 silent expression model was constructed, the expression of GSDMD, IL-1β and IL-18 protein (Fig. 6E) was significantly down-regulated. After TMP intervention, IL-1β and IL-18 decreased most significantly at the TMP concentration of 60 mg/L (Fig. 5C, D), and the effect was most significant at the TMP dose of 40 mg/L; the results indicated that the concentration of TMP 60 mg/L could significantly improve the inflammatory reaction of podocytes induced by high glucose.

## Discussion

*Chuanxiong* has anti-inflammatory, analgesic, anti-thrombotic, promoting vasodilation, asthma, anti-respiratory depression, anti-fibrosis, anti-obstructive diseases, anti-tumor, and other effects (Chen et al. 2018; Lin et al. 2022). In this study, phthalide compounds, TMP, and ferulic acid have the most extensive and robust effects among these active ingredients by using the Traditional Chinese Medicine (TCM) database. We used ConsensusClusterPlus to construct the correlation between the drug targets of *Chuanxiong* and cell pyroptosis. Besides, WGCNA was used to evaluate the degree of pyroptosis in the high glucose model. It is a hypothesis that *Chuanxiong* pharmacodynamic targets. e.g., DHCR24, CHI3L1, and CACNB2 can regulate DN’s progress. We found that DHCR24, CHI3L1, and CACNB2 can participate in pyroptosis-related signaling molecules, suggesting that *Chuanxiong* might potentially affect the pyroptotic state of cells, thereby inhibiting the development of the disease.

Furthermore, during the experiment, under the high glucose environment in vitro, we selected TMP, the active ingredient of *Chuanxiong*, for verification. Nephrin expression was concentration-dependent with TMP intervention; in our study, we detected the expression of macrophage resident inflammatory initiator IL-18 and found that the expression of IL-18 was associated with the activation of NF-κB signaling. After TMP intervention, the expressions of IL-18 and phosphorylated p65 were significantly down-regulated. This evidence indicates that the protective effect of *Chuanxiong* on DN is related to the improvement of the inflammatory state, of which the improvement of pyroptosis is a possible way.

DN is glucose-driven pathogenesis that occurs in the kidneys of people with diabetes. Pyroptosis occurs only when the excess glucose flux, advanced glycation end products (AGEs), and Reactive Oxygen Species (ROS) play critical roles, either as an inducer of the inflammasome or as a signaling pathway regulator (An et al. 2020; Lin et al. 2020; Liu et al. 2021; Tricò et al. 2022). Among the various inflammasomes that induce Pyroptosis, NLRP3 has been the most extensively investigated DN. High glucose can speed up the Toll-like receptor (TLR) 2 and TLR4, NLRP3 activate the upstream PRR (Wu et al. 2022). Likewise, Stimulation of TLR2 and TLR4 is critical for NLRP3 activation (Tan et al. 2022). Besides, as we all know, thioredoxin-interacting protein (TXNIP) belongs to the thioredoxin system. It can bind to thioredoxin (TRX), reducing the inhibitory effect of TRX on oxidative stress. TXNIP action is critical for NLRP3 activation and destructive cellular responses to multiple DAMPs, including pyroptosis (Vo et al. 2021). Diabetic kidneys show elevated TXNIP expression, and inhibition of TXNIP attenuates high glucose-induced renal injury, positioning TXNIP as a potential target for DN therapy (Dai et al. 2021b; Wang et al. 2022a). ROS has been recognized as a standard signal for NLRP3 activation and can activate NLRP3 through different pathways (Tang et al. 2017; Wu et al. 2021), of which the NF-κB pathway is the most widely studied. Activating the NF-κB signal mediated by ROS can lead to NLRP3 inflammasomes efficiently oligomerizing form. In DN, inhibition of the ROS/NF-κB/NLRP3 pathway appears to reduce inflammation and control the development of progressive kidney damage (Xu et al. 2021). In addition, endoplasmic reticulum (ER) stress is another trigger of the NLRP3 inflammasome. Hyperglycemia induces ER stress in podocytes, tubular epithelial cells, and mesangial cells (Hong et al. 2019). Upon activation, NLRP3 drives inflammatory responses by processing Caspase-1, IL-1β, and IL-18, contributing to DKD progression (Tang and Yiu 2020).

Recent research showed that the knockdown of NLRP3 could inhibit the growth and invasion of glioma cells (Xue et al. 2019), along with the decrease of IL-1β and NF-κB, indicating a positive correlation between NLRP3 and NF-κB. Blocking NF-κB attenuates IL-1β and NLRP3 overexpression and increases cell growth and invasion, and NLRP3 promotes glioma growth and invasion through IL-1β/ NF-κB P65 signaling (Wang et al. 2019). Besides, inhibiting NLRP3/caspase-1-mediated pyroptosis attenuates pathological changes in diabetic kidneys (Wang and Zhao 2021), suggesting its therapeutic potential in DN. Pyroptosis of different kidney-resident cells contributes to the development of DN.

Disruption of the glomerular endothelium alters the selective permeability of the glomerulus and occurs in the early stages of DN. Using confocal microscopy, researchers observed partial colocalization of NLRP3 or Cleaved caspase-1 in glomerular endothelial cells in kidney tissue sections from diabetic patients or mice (Shahzad et al. 2015). Studies have shown that high glucose intervention cuts off the N terminal of GSDMD, gradually forms membrane pores, and causes the release of pyrogen, a process that plays a role in inflammatory diseases, such as glomerular endothelial cells are damaged. Studies have shown that cell pyromorphite may be an effective mechanism of podocyte loss (Han et al. 2021). When Knocking down TXNIP or inhibiting NADPH oxidase, ROS negatively control NLRP3 activation and attenuate glucose-induced podocyte injury (Abais et al. 2013; Song et al. 2019). Besides, Liu et al. found that enhancing caspase-1 activity could elevate IL-1β and IL-18 levels in podocytes exposed to high glucose (Liu et al. 2019; Liu et al. 2018). Li et al. detected the N terminal of GSDMD in high glucose-damaged, a marker for diabetic podocytes (Li et al. 2020a). Thus, findings provide non-classical pyrolytic pathways can participate in podocyte injury.

TMP has the pharmacological properties of vasodilation and anti-inflammation. However, there is very little research on the pharmacological mechanism of *Chuanxiong*, especially for the protection of DN. The analysis of chemical and bioactive components of *Chuanxiong* originated in the 1930s. Some components belong to these three main chemical groups: alkaloids, phenolic acids, and phthalides. Among these components, TMP and ferulic acid are the most representative components of the function and structure of *Chuanxiong *(Wang et al. 2022c). In recent years, with the development of pharmacokinetics, the properties of *Chuanxiong* have also been widely studied. In vivo, TMP increases endothelial cells’ Ca^+^ concentrations, which may trigger eNOS expression, increase NO levels and improve vasospasm (Wang et al. 2022c; Zhou et al. 2020). This study potentially reveals that TMP can affect the expression of cellular inflammatory factors, thereby exerting a protective effect on vascular lesions, such as endothelial cell lesions. DN is a microvascular disease, and glomerular disease is the leading disease form. Endothelial cells and podocytes are involved in the regulation of glomerular function. In this study, based on a machine-learning algorithm, it was found that DHCR24, CHI3L1, and CACNB2, the pharmacodynamic targets of *Chuanxiong*, can regulate the expression network. In pyroptosis evaluation, DHCR24, CHI3L1, and CACNB2 are pyroptosis-related signal initiation molecules. Furthermore, TMP could affect podocytes’ pyroptosis and inflammatory cellular state. This evidence confirmed that *Chuanxiong* affects the occurrence of pyroptosis and then regulates the progression of inflammation, and the process may be related to the targets of *Chuanxiong* DHCR24, CHI3L1, and CACNB2.

In this study, a new method, a machine learning algorithm, is used to predict the pharmacological action of *Chuanxiong*. It is significant to explore the new mechanism of the pharmacological action of *Chuanxiong*. We anticipate more evidence, including new tools such as pharmacology and artificial intelligence, to support focus as a new drug target.

## Conclusion

Machine learning algorithm reveal targets of *Chuanxiong* DHCR24, CHI3L1, and CACNB2 can affects the occurrence of pyroptosis and then regulates the progression of inflammation.

## Data Availability

All data generated or analysed during this study are included in this published article [and its supplementary information files].

## References

[CR1] Abais J, Zhang C, Xia M, Liu Q, Gehr T, Boini K, Li P (2013). NADPH oxidase-mediated triggering of inflammasome activation in mouse podocytes and glomeruli during hyperhomocysteinemia. Antioxid Redox Signal.

[CR2] An X, Zhang Y, Cao Y, Chen J, Qin H, Yang L (2020). Punicalagin protects diabetic nephropathy by inhibiting pyroptosis based on TXNIP/NLRP3 pathway. Nutrients.

[CR3] Burdette B, Esparza A, Zhu H, Wang S (2021). Gasdermin D in pyroptosis. Acta Pharm Sinica B.

[CR4] Chen Z, Zhang C, Gao F, Fu Q, Fu C, He Y, Zhang J (2018). A systematic review on the rhizome of *Ligusticum chuanxiong* Hort. (Chuanxiong). Food Chemical Toxicol.

[CR5] Dai H, Hu W, Lin L, Wang L, Chen J, He Y (2021). Tubular decoy receptor 2 as a predictor of prognosis in patients with immunoglobulin A nephropathy. Clin kidney J.

[CR6] Dai X, Liao R, Liu C, Liu S, Huang H, Liu J, Jin T, Guo H, Zheng Z, Xia M, Ling W, Xiao Y (2021). Epigenetic regulation of TXNIP-mediated oxidative stress and NLRP3 inflammasome activation contributes to SAHH inhibition-aggravated diabetic nephropathy. Redox Biol.

[CR7] Han J, Zuo Z, Shi X, Zhang Y, Peng Z, Xing Y, Pang X (2021). Hirudin ameliorates diabetic nephropathy by inhibiting Gsdmd-mediated pyroptosis. Cell Biol Toxicol..

[CR8] Hong J, Bhat O, Li G, Dempsey S, Zhang Q, Ritter J, Li W, Li P (2019). Lysosomal regulation of extracellular vesicle excretion during d-ribose-induced NLRP3 inflammasome activation in podocytes. Biochimica et biophysica acta. Mol cell Res.

[CR9] Li F, Chen Y, Li Y, Huang M, Zhao W (2020). Geniposide alleviates diabetic nephropathy of mice through AMPK/SIRT1/NF-κB pathway. Eur J Pharmacol.

[CR10] Li M, Guo Q, Cai H, Wang H, Ma Z, Zhang X (2020). miR-218 regulates diabetic nephropathy via targeting IKK-β and modulating NK-κB-mediated inflammation. J Cell Physiol.

[CR11] Lin J, Cheng A, Cheng K, Deng Q, Zhang S, Lan Z, Wang W, Chen J (2020). New insights into the mechanisms of pyroptosis and implications for diabetic kidney disease. Int J Mol Sci.

[CR12] Lin J, Wang Q, Zhou S, Xu S, Yao K (2022). Tetramethylpyrazine: a review on its mechanisms and functions. Biomed Pharmacother.

[CR13] Liu B, Lu R, Li H, Zhou Y, Zhang P, Bai L, Chen D, Chen J, Li J, Yu P, Wu J, Liang C, Song J, Liu X, Zhou J (2019). Zhen-wu-tang ameliorates membranous nephropathy rats through inhibiting NF-κB pathway and NLRP3 inflammasome. Phytomedicine.

[CR14] Liu P, Zhang Z, Li Y (2021). Relevance of the pyroptosis-related inflammasome pathway in the pathogenesis of diabetic kidney disease. Front Immunol.

[CR15] Liu Y, Xu Z, Ma F, Jia Y, Wang G (2018). Knockdown of TLR4 attenuates high glucose-induced podocyte injury via the NALP3/ASC/Caspase-1 signaling pathway. Biomed Pharmacother.

[CR16] Rai U, Kosuru R, Prakash S, Tiwari V, Singh S (2019). Tetramethylpyrazine alleviates diabetic nephropathy through the activation of Akt signalling pathway in rats. Eur J Pharmacol.

[CR17] Rühl S, Shkarina K, Demarco B, Heilig R, Santos J, Broz P (2018). ESCRT-dependent membrane repair negatively regulates pyroptosis downstream of GSDMD activation.

[CR18] Shahzad K, Bock F, Dong W, Wang H, Kopf S, Kohli S, Al-Dabet M, Ranjan S, Wolter J, Wacker C, Biemann R, Stoyanov S, Reymann K, Söderkvist P, Groß O, Schwenger V, Pahernik S, Nawroth P, Gröne H, Madhusudhan T, Isermann B (2015). Nlrp3-inflammasome activation in non-myeloid-derived cells aggravates diabetic nephropathy. Kidney Int.

[CR19] Song S, Qiu D, Shi Y, Wang S, Zhou X, Chen N, Wei J, Wu M, Wu H, Duan H (2019). Thioredoxin-interacting protein deficiency alleviates phenotypic alterations of podocytes via inhibition of mTOR activation in diabetic nephropathy. J Cell Physiol..

[CR20] Tan H, Zhao Q, Chen L (2022). Penehyclidine hydrochloride suppresses inflammation response and reduces podocyte injury in diabetic nephropathy by targeting fibrinogen-like protein 2. Int Immunopharmacol.

[CR21] Tang S, Yiu W (2020). Innate immunity in diabetic kidney disease. Nat Rev Nephrol.

[CR22] Tang T, Lang X, Xu C, Wang X, Gong T, Yang Y, Cui J, Bai L, Wang J, Jiang W, Zhou R (2017). CLICs-dependent chloride efflux is an essential and proximal upstream event for NLRP3 inflammasome activation. Nat Commun.

[CR23] Tricò D, McCollum S, Samuels S, Santoro N, Galderisi A, Groop L, Caprio S, Shabanova V (2022). Mechanistic insights into the heterogeneity of glucose response classes in youths with obesity. A latent class trajectory approach. Diabetes care..

[CR24] Vo T, Lee C, Chiang Y, Chen Y, Yu Y, Tuan V, Wu C, Lee I (2021). Protective mechanisms of Taiwanese green propolis toward high glucose-induced inflammation via NLRP3 inflammasome signaling pathway in human gingival fibroblasts. J Periodontal Res.

[CR25] Wang A, Gong Y, Pei Z, Jiang L, Xia L, Wu Y (2022). Paeoniflorin ameliorates diabetic liver injury by targeting the TXNIP-mediated NLRP3 inflammasome in db/db mice. Int Immunopharmacol.

[CR26] Wang A, Zhang D, Liu J, Yan H, Zhang P, Yuan H, Ma X (2022). Guanxinning injection combined with ischemic postconditioning attenuate myocardial ischemic reperfusion injury in chronic renal failure rats by modulating mitochondrial dynamics. Front Cardiovasc Med.

[CR27] Wang J, Wang L, Zhou H, Liang X, Zhang M, Tang Y, Wang J, Mao J (2022). The isolation, structural features and biological activities of polysaccharide from *Ligusticum chuanxiong*: a review. Carbohydr Polym.

[CR28] Wang J, Zhao S (2021). LncRNA-antisense non-coding RNA in the INK4 locus promotes pyroptosis via miR-497/thioredoxin-interacting protein axis in diabetic nephropathy. Life Sci.

[CR29] Wang R, Wu Y, An D, Ma P, Guo Y, Tang L (2020). Case report: glucocorticoids combined with immunosuppressant in the treatment of acromegaly complicated with focal segmental glomerulosclerosis. Front Med.

[CR30] Wang Y, Huang J, Chen W, Wang R, Kao M, Pan Y, Chan S, Tsai K, Kung H, Lin K, Wang L (2019). Dysregulation of cystathionine γ-lyase promotes prostate cancer progression and metastasis. EMBO Rep.

[CR31] Wonnacott A, Denby L, Coward R, Fraser D, Bowen IJA (2022). MicroRNAs and their delivery in diabetic fibrosis. Adv Drug Deliv Rev.

[CR32] Wu C, Liu J, Li Y, Wang N, Yan Q, Jiang Z (2022). Manno-oligosaccharides from cassia seed gum ameliorate inflammation and improve glucose metabolism in diabetic rats. Food Funct.

[CR33] Wu K, Long K, Lin H, Siu P, Hoo R, Ye D, Xu A, Cheng K (2021). The APPL1-Rab5 axis restricts NLRP3 inflammasome activation through early endosomal-dependent mitophagy in macrophages. Nat Commun.

[CR34] Xu X, Huang X, Zhang L, Huang X, Qin Z, Hua F (2021). Adiponectin protects obesity-related glomerulopathy by inhibiting ROS/NF-κB/NLRP3 inflammation pathway. BMC Nephrol.

[CR35] Xue L, Lu B, Gao B, Shi Y, Xu J, Yang R, Xu B, Ding P (2019). NLRP3 promotes glioma cell proliferation and invasion via the interleukin-1β/NF-κB p65 signals. Oncol Res.

[CR36] Yang J, Yu X, Hu N, Su T (2022). Clinical and pathological features of renal presentations in polycythemia vera. Am J Med Sci.

[CR37] Zhang Q, Xu S, Qian J, Yang L, Chen P, Wang Y, Hu X, Zhang Y, Luo W, Liang G (2022). Pharmacological inhibition of MyD88 suppresses inflammation in tubular epithelial cells and prevents diabetic nephropathy in experimental mice. Acta Pharmacol Sin.

[CR38] Zhang Y, He L, Ma C, Wang C, Zhou H, Guo C, Gong L, Wan Y, Peng C, Li Y (2022). Research progress on the pharmacy of tetramethylpyrazine and its pharmacological activity in cardiovascular and cerebrovascular diseases. J Pharm Pharmacol.

[CR39] Zhou Q, Chen S, Li H, Yang B, Chen T, Hu T, Yin D, He H, He M (2020). Tetramethylpyrazine alleviates iron overload damage in vascular endothelium via upregulating DDAHII expression. Toxicol In Vitro.

[CR40] Zhu T, Fang B, Meng X, Zhang S, Wang H, Gao G, Liu F, Wu Y, Hu J, Sun G, Sun X (2022). GinkgoFolium extract and tetramethylpyrazine sodium chloride injection (Xingxiong injection) protects against focal cerebral ischaemia/reperfusion injury via activating the Akt/Nrf2 pathway and inhibiting NLRP3 inflammasome activation. Pharm Biol.

